# Effect of Particle Size on Pickering Emulsion Stability Under Different Homogenization Methods

**DOI:** 10.1021/acs.langmuir.6c01064

**Published:** 2026-06-15

**Authors:** Lin Chen, Patrícia Figueiredo, Alaa Mahran, Ville A. Lovikka, Jessica M. Rosenholm, Maarit H. Lahtinen, Kirsi S. Mikkonen

**Affiliations:** † Department of Food and Nutrition, Faculty of Agriculture and Forestry, University of Helsinki, FIN-00014 Helsinki, Finland; ‡ Pharmaceutical Sciences Laboratory, Faculty of Science and Engineering, Åbo Akademi University, Turku 20520, Finland; § Department of Pharmaceutics, Faculty of Pharmacy, Assiut University, Assiut 71526, Egypt; ∥ Helsinki Institute of Sustainability Science (HELSUS), University of Helsinki, FIN-00014 Helsinki, Finland

## Abstract

Pickering emulsions have attracted significant interest due to their superior stability compared to surfactant-stabilized emulsions. Particle size is a key parameter in the stabilization of Pickering emulsions. However, a fundamental understanding of how particle size influences emulsion stabilization across different homogenization methods remains unclear. In this study, the effects of silica nanoparticle size on Pickering emulsion formation and stability were investigated under different processing methods, including rotor–stator homogenization, ultrasonication, and high-pressure homogenization. Emulsion structure and stability were evaluated using droplet size analysis, interfacial adsorption measurements, ζ-potential characterization, and Turbiscan stability analysis. Multivariate Partial Least Squares (PLS) regression was further applied to assess the relative importance of particle size. The results show that the effect of particle size on emulsion stability strongly depends on the processing method. Under rotor–stator homogenization, stability was governed by a balance between droplet size reduction and particle sedimentation, resulting in optimal stability for intermediate-sized nanoparticles. Under ultrasonication, smaller nanoparticles enhanced stability primarily through increased interfacial adsorption. In contrast, for high-pressure homogenization, particle size effects on droplet structure were largely masked, and stability was mainly constrained by limited coalescence during storage and sedimentation of larger particles. These findings show that particle size influences Pickering emulsion stability through distinct mechanisms under different homogenization methods, providing guidance for the rational design of particle-stabilized emulsions.

## Graphical abstract

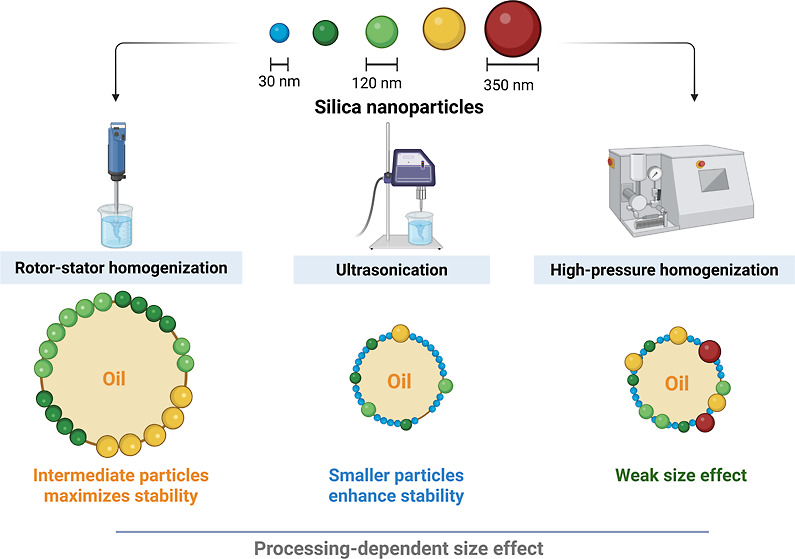

## Introduction

Pickering emulsions, stabilized by solid particles rather than conventional surfactants, have gained increasing attention due to their enhanced stability and potential in a wide range of applications, including food,^1^ cosmetics,^2^ and pharmaceuticals.^3^ The phenomenon of solid-stabilized emulsions was initially observed and described by Ramsden^4^ and Pickering.^5^ Subsequent studies by Binks^6^ and Chevalier^7^ further delved into the stabilization mechanism and formation of Pickering emulsions. Advances in material science have expanded the range of particles used for Pickering emulsions, including inorganic particles,^8–10^ synthetic polymer particles,^11,12^ and natural biopolymers.^13,14^ Among these, silica nanoparticles are widely employed owing to their tunable surface properties and availability across a broad size range, making them a suitable model system for studying particle-stabilized emulsion behavior.^2,15^

The superior stability of Pickering emulsions, compared to surfactant-stabilized emulsions, arises from high energy barrier associated with particle detachment from the oil–water interface.^7^ As a result, emulsion stability is governed by both interfacial and structural factors, including particle properties, droplet size distribution, and their evolution during processing and storage.^16^ Particle properties, including size,^17^ wettability,^18,19^ surface charge,^20^ shape,^21^ and concentration,^22,23^ are key parameters governing Pickering emulsion formation and stability. While the roles of particle wettability and concentration have been extensively investigated, the influence of particle size remains poorly understood. Only a few studies have examined the role of particle size in Pickering emulsions, and these studies are mostly limited to a single processing method or similar energy input.^17,24^ In practice, emulsions are prepared using different processing methods to meet diverse application requirements, as different applications demand emulsions with specific droplet sizes, stability levels, and structural characteristics. Common techniques such as rotor–stator homogenization, ultrasonication, high-pressure homogenization, membrane emulsification, and microfluidic emulsification are widely used in emulsion preparation.^25,26^ Rotor-stator homogenization is a simple and accessible baseline method for dispersing immiscible phases by mechanical shear.^27^ Ultrasonication is commonly employed to produce finer droplets through acoustic cavitation, and the localized high pressures and intense shear forces contribute to efficient particle dispersion and adsorption at the interface.^28^ High-pressure homogenization is widely used in industrial processing. It generates fine and relatively uniform droplets through high-pressure fluid dynamics and intense shear forces.^29^ Those homogenization methods generate droplets and interfaces through distinct mechanisms and energy dissipation pathways, which can influence droplet breakup, particle dispersion, and adsorption at the oil–water interface. Consequently, differences in processing conditions are often accompanied by variations in droplet size distribution, interfacial particle coverage, and the destabilization mechanisms during storage. It remains unclear whether particle size exerts a consistent effect on emulsion stability across different homogenization conditions. Therefore, a systematic comparison across distinct processing pathways is necessary to clarify the mechanistic role of particle size in droplet formation and stabilization.

In this study, we systematically investigated how silica nanoparticle size influences Pickering emulsion formation and stability under rotor–stator homogenization, ultrasonication, and high-pressure homogenization. These three methods were selected as they are widely used in both laboratory and industrial settings and produce emulsions with distinct droplet characteristics. Silica nanoparticles with mean diameters ranging from 30 to 350 nm were used as stabilizers. Emulsion structure and stability were characterized through droplet size analysis, interfacial adsorption measurements, ζ-potential characterization, and Turbiscan stability analysis. In addition, PLS regression was applied to study the correlation between particle size, droplet size, and emulsion stability. This study aims to clarify how particle size influences Pickering emulsion stability under different processing methods. Addressing this question is important for establishing a reliable understanding of particle size effects, guiding the selection of particle size and processing methods for practical formulations.

## Materials and Methods

### Materials

Commercial aqueous colloidal dispersions of silica nanoparticles, Levasil CS30-324 P (30%, by volume; pH 10.0) and Levasil CS50-34 P (50%, by volume; pH 9.8), were supplied by Nouryon (Amsterdam, Netherlands). Laboratory-prepared colloidal silica nanoparticles were synthesized using the Stöber method.^30^ For clarity and ease of reference, the silica nanoparticles were named according to their origin and hydrodynamic average size: CS30, CS100, LS120, LS150, and LS350. Commercial silica samples are denoted as CS, while laboratory-prepared samples are abbreviated as LS (Table 1). Oleic acid (>90%), hexadecane (>98%) were purchased from Sigma-Aldrich. All solutions and dispersions were prepared using Milli-Q water.

**Table 1:** Abbreviations of Silica Nanoparticles and Emulsion Preparing Methods

abbreviations	full names
CS30	commercial silica with a mean hydrodynamic diameter of 30 nm
CS100	commercial silica with a mean hydrodynamic diameter of 100 nm
LS120	lab-prepared silica with a mean hydrodynamic diameter of 120 nm
LS150	lab-prepared silica with a mean hydrodynamic diameter of 150 nm
LS350	lab-prepared silica with a mean hydrodynamic diameter of 350 nm
UT	ultra-Turrax, rotor-stator homogenization
US	ultrasonicator, ultrasound homogenization
MF	microfluidizer, high-pressure homogenization

### Silica Nanoparticle Modification and Characterization

The silica nanoparticles were modified using oleic acid according to previous research with some modifications.^31^ Briefly, the silica nanoparticle suspension was diluted to 10 mg/mL, and the pH was adjusted to 7 using HCl (1 mol/L). The samples were ultrasonicated (Branson 450 Digital Sonifer, Marshall Scientific, Hampton, USA) for 2 min at 30% amplitude to avoid aggregation. Oleic acid was added to water at the concentration of 2.5 mM, and then the mixture was ultrasonicated for 10 min at 30% amplitude to enable the dispersion of oleic acid in water as micrometer-sized droplets. The silica nanoparticle suspension and oleic acid dispersion were mixed at the ratio of 1:1 (w/w). The resulting sample was immersed in an ice bath and sonicated at 30% amplitude using pulse mode (2.5 s pulses with 0.5 s pauses) for 10 min for the adsorption of oleic acid at the silica interface. The lab-synthesized silica samples were first centrifuged and washed twice to remove ethanol and then redispersed in water for the modification with the same procedure.

The average size, polydispersity index (PDI), and ζ-potential of silica nanoparticles before and after modification was measured by dynamic light scattering (DLS), using a Malvern Zetasizer (Malvern Panalytical Ltd., Malvern, UK). The samples were diluted at a concentration of 0.5 mg/mL silica content for the measurement.

### Contact Angle

The wettability of silica nanoparticles was evaluated by contact angle measurements using an optical tensiometer (KSV CAM 200, Biolin Scientific, Finland). Thin films of silica nanoparticles were prepared via adsorption according to the method described by Farooq et al. with slight modifications.^32^ Glass slides (1.5 cm × 1.5 cm) were used as substrates. The slides were immersed in 1 M NaOH solution for 15 s, rinsed with Milli-Q water, and dried under nitrogen. Then, the substrates were treated with plasma (Zepto, Diener Electronic, Germany) for complete surface cleaning and enhanced hydrophilicity, facilitating subsequent adsorption. An anchoring layer of poly-l-lysine (PLL) was first adsorbed onto the substrates by immersing them in PLL solution for 12 h. After rinsing off excess PLL, the substrates were exposed to silica nanoparticle dispersions (1 g/L) for 30 min to allow particle adsorption. The substrates were then rinsed with Milli-Q water and dried under nitrogen.

Contact angles were measured using the sessile drop method. A droplet of deionized water (∼6.5 μL) was deposited onto the surface, and images were recorded every second for 1 min. The contact angle was determined by fitting the droplet profile using the Young–Laplace model. All measurements were performed at room temperature, and at least five independent measurements were conducted for each sample.

### Emulsion Preparation

Emulsions with different droplet sizes were prepared using different techniques including rotor-stator homogenization, ultrasonication, and high-pressure homogenization. Hexadecane was added to the modified silica nanoparticle suspension (5 mg/mL) to reach the concentration of 5 wt %. According to the processing method, the emulsions were divided into three groups. UT group, the mixture was stirred using Ultra-Turrax (T-18 basic, IKA, Staufen, Germany) equipped with an S25N-18G head at 10,000 rpm for 2 min to form emulsions. US group, the mixture was primarily stirred using Ultra-Turrax (10,000 rpm, 2 min) and then ultrasonicated in an ice bath for 2 min at 30% amplitude to form emulsion. MF group, the mixture was stirred using Ultra-Turrax (10,000 rpm, 2 min), followed by Microfluidizer (M-100Y, Microfluidics, Westwood, MA, USA) at 80 MPa for 4 cycles to prepare emulsions with fine droplets. The abbreviations of samples and emulsion preparation methods are listed in Table 1.

### Droplet Size and Distribution

The droplet size distribution of the emulsions after preparation (day 0) and storage for 1 month (day 28) was assessed using a Mastersizer Hydro 3000 SM (Malvern Instruments Ltd., Worcestershire, UK) employing Mie theory.^33^ Refractive indexes of 1.33 and 1.434 were used for the water and hexadecane, respectively. Before sampling, emulsions were gently mixed by turning upside down and sampled at half the height of the emulsion volume.

### ζ-Potential

The ζ-potential of oil droplets in emulsions were measured using Zetasizer. The samples were diluted 1:100 in Milli-Q water to avoid multiple scattering effects.

### Silica Adsorption on o/w Interface

The emulsion (10 g) was partitioned using centrifugation (16,099 × g, 15 min, 4 °C) (Sorvall LYNX 6000, Thermo Scientific, USA) to separate the silica nanoparticles dispersed in the continuous phase from the ones at the oil/water interface. The aqueous phase was collected, lyophilized, and subsequently dried in a vacuum oven at 40 °C for 3 days to assess the quantity of silica nanoparticles remaining in the continuous phase. The silica content adsorbed on the droplet interface was determined by subtracting the amount of silica remaining in the aqueous phase from the total mass.

### Turbiscan Stability

The physical stability of emulsions during storage at 25 °C over 1 month was determined by a Turbiscan analyzer (Formulations, France). Approximately 20 mL of freshly made emulsions were transferred to transparent glass vials and they were kept undisturbed at room temperature with natural light during storage. The kinetic stability of emulsions during storage was monitored using Turbiscan stability index (TSI) analysis.

### Data Analysis

Data of emulsion droplet size, ζ-potential were presented as means ± standard deviations (SD) from three replicates. The analysis of variance (ANOVA) was performed with SPSS (Version 12.0, SPSS Inc., IL, USA) and the Tukey test was used to determine significant differences (*P* < 0.05). The correlation between particle size and the stability of emulsion was investigated by the software SIMCA 17.0 (Satorius, German). PLS regression algorithm was applied to model the relationship between the predictor variables and the response variable. In our case, the stability (TSI-day28) of the Pickering emulsion is set as the Y variable, while particle size, emulsion size information, ζ-potential, and silica adsorption were set as X variables. The emulsions prepared with different processing methods (UT, US, and MF) were analyzed using different models. A critical point in developing the SIMCA model is to determine the optimal number of principal components (PCs) involved in the model. The root-mean-square error of cross-validation (RMSECV) was calculated, which represents the average difference between predicted and observed values. The optimal number of PCs was chosen based on the lowest RMSECV. The plots of the Observed stability vs Predicted stability, and the Biplots were generated to show the relationships between emulsion stability and variables.^34^

## Results and Discussions

### Surface Modification of Silica Nanoparticles

Bare silica nanoparticles are highly hydrophilic and therefore have limited ability to stabilize emulsions through interfacial adsorption.^35^ In this study, oleic acid was used to modify the surface of silica nanoparticles in order to adjust their wettability. Oleic acid was selected because its long hydrophobic alkyl chain can effectively increase particle hydrophobicity while maintaining good particle dispersibility.

Contact angle measurements show a clear increase in hydrophobicity after oleic acid modification (Figure 1a). This change in wettability facilitates particle adsorption at the oil–water interface, which is a key requirement for Pickering emulsion stabilization. For unmodified silica, CS30 exhibit lower contact angles, indicating higher hydrophilicity. This can be attributed to their higher specific surface area and greater density of exposed silanol groups. After oleic acid modification, CS30 still shows lower contact angle values compared to larger particles. This is likely due to its significantly higher surface area, resulting in a lower effective surface coverage of oleic acid and the persistence of hydrophilic surface sites. As shown in Figure 1b, oil–water mixtures containing oleic acid-modified silica nanoparticles formed more stable emulsions compared to systems containing either unmodified silica nanoparticles or oleic acid alone. The interfacial tension of the modified nanoparticle dispersion against hexadecane was measured to assess surface activity (Table S1). The interfacial tension between water and hexadecane decreased by 10 mN/m with modified silica nanoparticles, indicating oleic acid may contribute to interfacial tension reduction and facilitate droplet formation. However, emulsions prepared using oleic acid alone were unstable. Hexadecane rapidly migrated upward and formed a separate oil layer, leaving only a very thin emulsion layer after 1 day. In contrast, emulsions stabilized by modified silica nanoparticles remained stable for at least 3 months. These results suggest that the long-term stability of the emulsions is mainly governed by Pickering stabilization from silica nanoparticles. The interaction between oleic acid and silica is mainly attributed to hydrogen bonding between the carboxyl groups of oleic acid and silanol groups of silica. The adsorption of oleic acid might be influenced by the emulsification process, particularly under high-energy conditions. In our study, the contact angles of the modified silica nanoparticles were measured before and after ultrasonication and high-pressure homogenization. No significant differences were observed, suggesting that the surface wettability was largely preserved during processing (Figure S1, Supporting Information).

**Figure 1:**
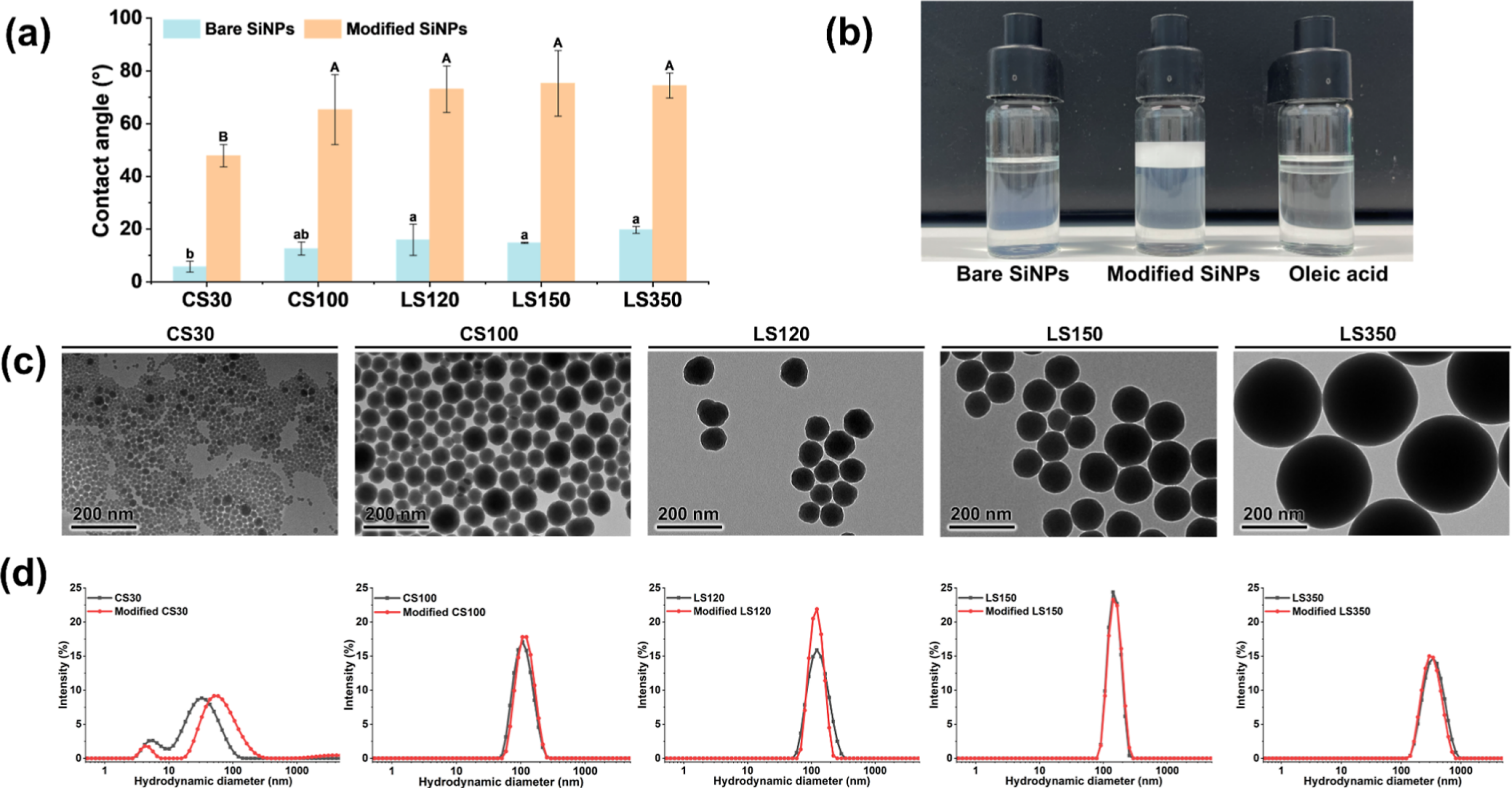
(a) Contact angle of silica nanoparticles measured at the water–air interface. (b) Photographs of oil–water mixtures containing bare silica nanoparticles, oleic acid–modified silica nanoparticles, and oleic acid alone. (c) TEM images showing the morphology of dried silica nanoparticles. (d) Size distributions of silica nanoparticles in water measured by DLS before and after oleic acid modification.

The morphologies of the five silica nanoparticle samples were examined by TEM, confirming their spherical shape and distinct particle sizes (Figure 1c). To obtain representative particle size distributions, we also performed DLS measurements (Figure 1d). The results showed that oleic acid modification did not significantly change the particle size distribution or induce noticeable aggregation. The average particle size and PDI of modified silica nanoparticles are shown in Table 2. The similar particle size after modification indicates that modified silica nanoparticles provide a suitable model system for investigating the effects of particle size on Pickering emulsion formation and stability.

**Table 2:** Hydrodynamic Size, PDI, and ζ-Potential of Silica Nanoparticles^a^

sample	hydrodynamic diameter/nm	PDI	ζ-potential of bare particles/mV	ζ-potential of modified particles/mV
CS 30	32.4 ± 7.7	0.438 ± 0.006	–53.4 ± 1.4	–63.2 ± 7.9
CS 100	107.7 ± 3.9	0.105 ± 0.010	–58.3 ± 0.6	–80.6 ± 1.4
LS 120	125.1 ± 1.1	0.034 ± 0.025	–48.7 ± 1.1	–59.1 ± 0
LS 150	154.1 ± 0.3	0.045 ± 0.003	–52.2 ± 1.9	–76.4 ± 7.8
LS 350	355.5 ± 4.4	0.064 ± 0.052	–58.2 ± 1.0	–72.5 ± 1.5

a Silica nanoparticles were dispersed in Milli-Q water at a concentration of 0.5 mg/mL.

After modification, all samples exhibited more negative ζ-potential values than the bare silica nanoparticles (Table 2). The more negative ζ-potential observed after oleic acid treatment suggests that the particle surface was altered by the treatment. This change may be related to presence of adsorbed oleic acid at the particle surface, changes in the dissociation behavior of surface silanol groups, and modifications of the electrical double layer. Since the adsorption amount and surface coverage of oleic acid were not directly quantified in the present study, the relative contribution of each factor cannot be determined conclusively. From the results, we observed that CS100 showed a larger decrease in ζ-potential than CS30, while LS150 and LS350 exhibited larger decreases compared to LS120. This trend could be explained by differences in specific surface area. Smaller particles possessed a higher specific surface area, resulting in a lower effective surface coverage of oleic acid. Consequently, the modification had a weaker influence on the surface charge of smaller particles, leading to a smaller change in ζ-potential. These results were partly consistent with the contact angle measurements shown in Figure 1a, where CS30 exhibited higher hydrophilicity.

### Effect of Particle Size on Emulsion Droplet Size

Based on the established model system, the effects of particle size on emulsion droplet size and distribution were investigated under different homogenization methods. Pickering emulsions were prepared by rotor–stator homogenization (Ultra-Turrax, UT), ultrasonic homogenization (Ultrasonicator, US), and high-pressure homogenization (Microfluidizer, MF). The corresponding droplet size distributions determined by static light scattering are shown in Figure 2.

**Figure 2:**
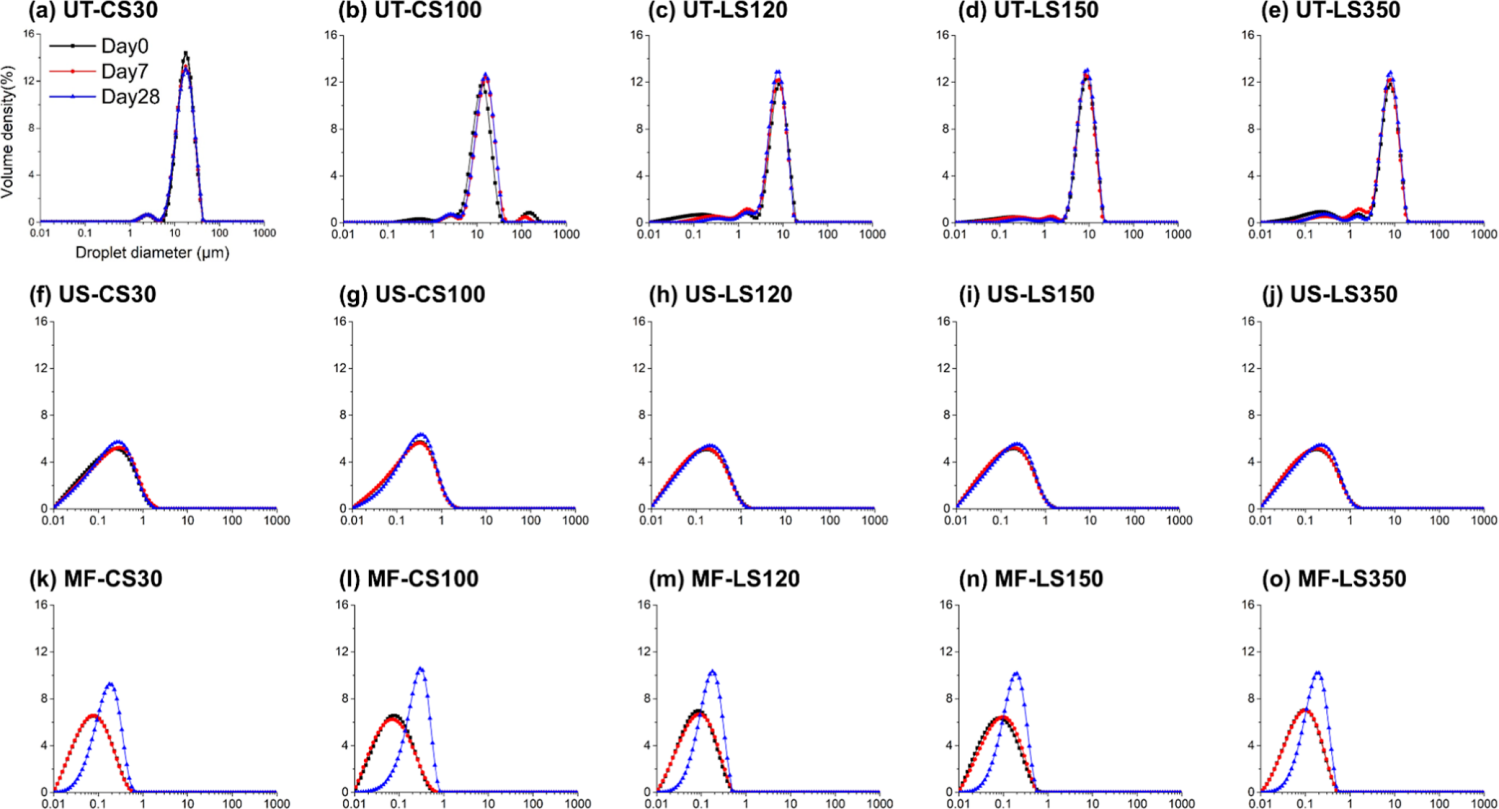
Size distribution of oil droplets in Pickering emulsions with different silica nanoparticles and processing methods (UT, US, MF). Fresh emulsions (day 0, black lines), samples after 7 days (red lines) and 28 days (blue lines) were compared. All samples contained 5 mg/mL silica nanoparticles.

Under UT conditions (Figure 2a–e), emulsions stabilized by silica nanoparticles of different sizes exhibited broad droplet size distributions spanning from 0.01 to 10 μm. During storage, smaller droplets gradually aggregated into larger ones, indicating a limited resistance to droplet aggregation. The corresponding droplet size parameters are summarized in Table 3. Under these conditions, particle size exerted a clear influence on the resulting droplet size. Specifically, an increase in silica nanoparticle size was associated with a decrease in droplet size in UT-prepared emulsions. Previous studies have commonly reported that smaller particles tend to produce smaller emulsion droplets.^17,26^ The observed trend deviates from the particle–droplet size relationship commonly reported in the literature. The average droplet size decreased from 13.3 to 2.2 μm as the silica nanoparticle size increased from 30 to 350 nm. According to the contact angle measurements (Figure 1a), CS30 exhibited a lower contact angle and higher hydrophilicity than the larger particles. The higher hydrophilicity might limit its interfacial adsorption during emulsification, resulting in larger droplet sizes. A similar behavior has been reported for Pickering emulsions prepared by rotor–stator homogenization, where starch particles with sizes between 100 and 220 nm generated smaller oil droplets and improved emulsion stability compared to particles below 100 nm.^24^ This behavior may be related to the balance between droplet breakup and particle adsorption during rotor–stator homogenization. Larger particles diffuse more slowly^36^ and may therefore delay interfacial coverage at the early stage of emulsification, allowing droplets to experience further deformation and breakup under high shear. In addition, larger particles may contribute to stronger local flow disturbances within the rotor–stator zone, which could further promote droplet breakup before effective interfacial stabilization is established. In contrast, smaller nanoparticles are likely to adsorb more rapidly at the oil–water interface, limiting additional droplet fragmentation.

**Table 3:** Droplet Size Parameters of Pickering Emulsions After 28 days of Storage Prepared Under Different Homogenization Conditions^a^ ^,^ ^b^ ^,^ ^c^

group	sample	D [3,2] (μm)	D [4,3] (μm)	Dv (10) (μm)	Dv (50) (μm)	Dv (90) (μm)	specific surface area (m^2^/kg)
UT	UT-CS30	13.3 ± 0.6^A^	17.9 ± 0.3^A^	9.5 ± 0.7^A^	9.5 ± 0.7^A^	17.0 ± 0.4^A^	5.9 ± 0.2(×10^2^)^C^
	UT-CS100	10.7 ± 0.3^B^	14.2 ± 1.1^B^	6.9 ± 0.3^B^	6.9 ± 0.3^B^	13.5 ± 1.0^B^	7.3 ± 0.2(×10^2^)^C^
	UT-LS120	3.6 ± 0.0^C^	7.3 ± 0.0^C^	3.1 ± 0.0^CD^	3.1 ± 0.0^C^	7.1 ± 0.0^C^	2.2 ± 0.0(×10^3^)^B^
	UT-LS150	4.1 ± 0.1^C^	8.6 ± 0.3^C^	4.3 ± 0.1^C^	4.3 ± 0.1^C^	8.2 ± 0.2^C^	1.9 ± 0.0(×10^3^)^B^
	UT-LS350	2.2 ± 0.4^D^	7.4 ± 0.5^C^	2.6 ± 1.2^D^	2.6 ± 1.2^C^	7.2 ± 0.4^C^	3.7 ± 0.5(×10^3^)^A^
US	US-CS30	0.11 ± 0.01^B^	0.30 ± 0.02^B^	0.04 ± 0.01^B^	0.22 ± 0.02^B^	0.68 ± 0.05^B^	7.2 ± 0.6(×10^4^)^B^
	US-CS100	0.13 ± 0.00^A^	0.34 ± 0.00^A^	0.06 ± 0.00^A^	0.26 ± 0.00^A^	0.74 ± 0.00^A^	5.9 ± 0.1(×10^4^)^C^
	US-LS120	0.08 ± 0.01^C^	0.22 ± 0.01^C^	0.03 ± 0.00^C^	0.15 ± 0.01^C^	0.52 ± 0.03^C^	9.5 ± 0.6(×10^4^)^A^
	US-LS150	0.08 ± 0.00^C^	0.23 ± 0.00^C^	0.03 ± 0.00^C^	0.15 ± 0.00^C^	0.52 ± 0.00^C^	9.5 ± 0.1(×10^4^)^A^
	US-LS350	0.08 ± 0.00^C^	0.22 ± 0.00^C^	0.03 ± 0.00^C^	0.15 ± 0.00^C^	0.51 ± 0.00^C^	9.7 ± 0.1(×10^4^)^A^
MF	MF-CS30	0.12 ± 0.01^A^	0.18 ± 0.01^A^	0.06 ± 0.01^A^	0.16 ± 0.01^A^	0.33 ± 0.01^A^	6.5 ± 0.4(×10^4^)^A^
	MF-CS100	0.12 ± 0.00^A^	0.19 ± 0.01^A^	0.06 ± 0.02^A^	0.17 ± 0.01^A^	0.33 ± 0.05^A^	6.8 ± 0.5(×10^4^)^A^
	MF-LS120	0.12 ± 0.00^A^	0.17 ± 0.00^A^	0.06 ± 0.00^A^	0.15 ± 0.00^A^	0.30 ± 0.00^A^	6.6 ± 0.0(×10^4^)^A^
	MF-LS150	0.12 ± 0.01^A^	0.17 ± 0.01^A^	0.06 ± 0.00^A^	0.16 ± 0.01^A^	0.31 ± 0.01^A^	6.5 ± 0.3(×10^4^)^A^
	MF-LS350	0.12 ± 0.01^A^	0.17 ± 0.01^A^	0.06 ± 0.01^A^	0.15 ± 0.01^A^	0.30 ± 0.01^A^	6.8 ± 0.5(×10^4^)^A^

a Statistical significance of particle size effects within each homogenization method was evaluated by one-way ANOVA (*p* < 0.05). For each homogenization method, different superscript letters within the same column indicate statistically significant differences among emulsions prepared with different particle sizes.

b *D*[3,2] represents the surface-weighted mean diameter, and *D*[4,3] represents the volume-weighted mean diameter. *D*_v_(10), *D*_v_(50), and *D*_v_(90) denote the cumulative volume percentiles. The specific surface area (SSA) was calculated as SSA = 6/(ρ*D­[3,2]), where ρ is the density of hexadecane.

c The measured size may be influenced by the presence of unadsorbed silica nanoparticles.

In contrast, under US processing (Figure 2f–j), emulsions exhibited more homogeneous droplet size distributions and showed minimal changes during storage. In this case, the influence of particle size on droplet size was less pronounced, suggesting that the high energy input during ultrasonication reduced the sensitivity of droplet formation to particle size. Although minor differences were observed between emulsions stabilized by commercial and lab-synthesized silica nanoparticles, no consistent particle size–dependent trend was identified.

For MF-prepared emulsions (Figure 2k–o), fine droplets were initially obtained for all particle sizes. However, an increase in droplet size was observed after one month of storage, which can be attributed to limited coalescence occurring during storage. Although high-pressure homogenization initially produces very small droplets, the interfacial area may be too large to be fully covered by silica nanoparticles, allowing droplets to coalesce until sufficient interfacial coverage is reached.^37^ A similar phenomenon has been reported for Pickering emulsions stabilized by silica microrods.^38^ Despite this change, emulsions stabilized by silica nanoparticles of different sizes exhibited comparable droplet sizes after storage, indicating that particle size had a limited effect on droplet size under high pressure homogenization.

Overall, the influence of particle size on emulsion droplet size was strongly dependent on the homogenization method. Particle size effects were most evident under UT conditions, whereas they became weak under US and MF processing. These results indicate that high-energy homogenization can override particle size effects during droplet formation, thereby reducing particle size–dependent differences in emulsion structure.

### Interfacial Adsorption of Silica Nanoparticles and ζ-Potential

The fraction of silica nanoparticles adsorbed at the oil–water interface is shown in Figure 3. Clear differences in interfacial adsorption were observed among the emulsions prepared using different homogenization methods. For UT-prepared emulsions, the adsorption of silica nanoparticles ranged from 10.7% to 24.1%. In comparison, higher adsorption levels were obtained for emulsions prepared by US (17.9%–48.6%) and MF (23.4%–53.2%), indicating that higher energy input promotes particle adsorption at the interface.

**Figure 3:**
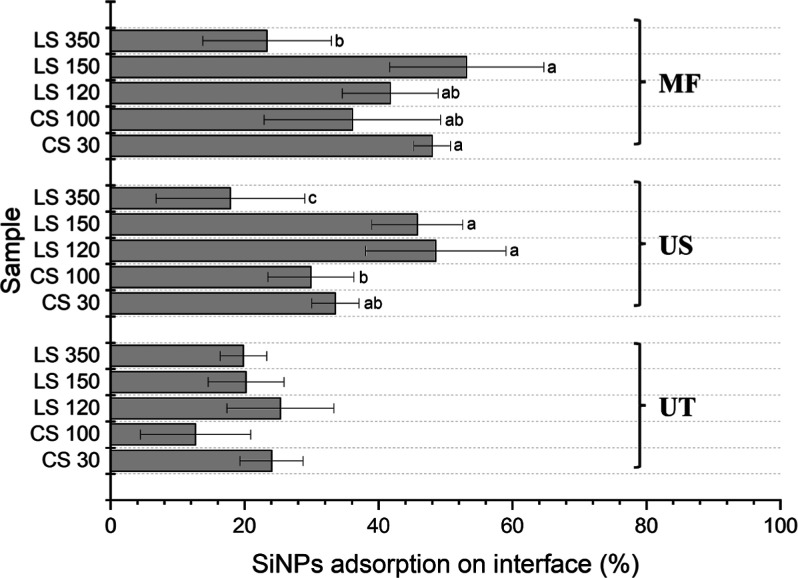
Content of silica adsorption at the oil–water interface. No significant differences were observed among UT samples (one-way ANOVA, *p* > 0.05).

The interfacial adsorption of particles is strongly influenced by particle size, wettability, and homogenization method. Within the same homogenization method, emulsions stabilized by smaller silica nanoparticles generally exhibited higher interfacial adsorption. For commercial silica nanoparticles, CS30 consistently showed greater adsorption than CS100. A similar trend was observed for lab-synthesized silica nanoparticles, where LS120 and LS150 displayed higher adsorption levels than LS350 under both US and MF conditions. These results suggest that smaller nanoparticles are more effective in covering the oil–water interface, likely due to their higher number density and more efficient packing at the droplet surface.

In addition to interfacial adsorption, the electrostatic characteristics of the droplet interface were assessed by ζ-potential measurements (Figure 4). All emulsions exhibited consistently negative ζ-potential values, confirming the presence of silica nanoparticles at the oil–water interface. It should be noted that the measured ζ-potential reflects the overall electrostatic response of the dispersion and may include contributions from both adsorbed and nonadsorbed silica nanoparticles present in the continuous phase. Therefore, it does not directly quantify the extent of particle adsorption. The magnitude of the ζ-potential was comparable across emulsions prepared by UT, US, and MF, indicating similar electrostatic characteristics of the droplet interface regardless of homogenization method. During storage, UT- and US-prepared emulsions showed a slight increase in the absolute ζ-potential, whereas MF-prepared emulsions exhibited a small decrease. This decrease is consistent with the increase in droplet size observed for MF emulsions during storage, which reduces the available interfacial area for particle adsorption and weakens electrostatic repulsion. Although some variations in ζ-potential were observed with particle size and particle type, no systematic particle size–dependent trend was identified.

**Figure 4:**
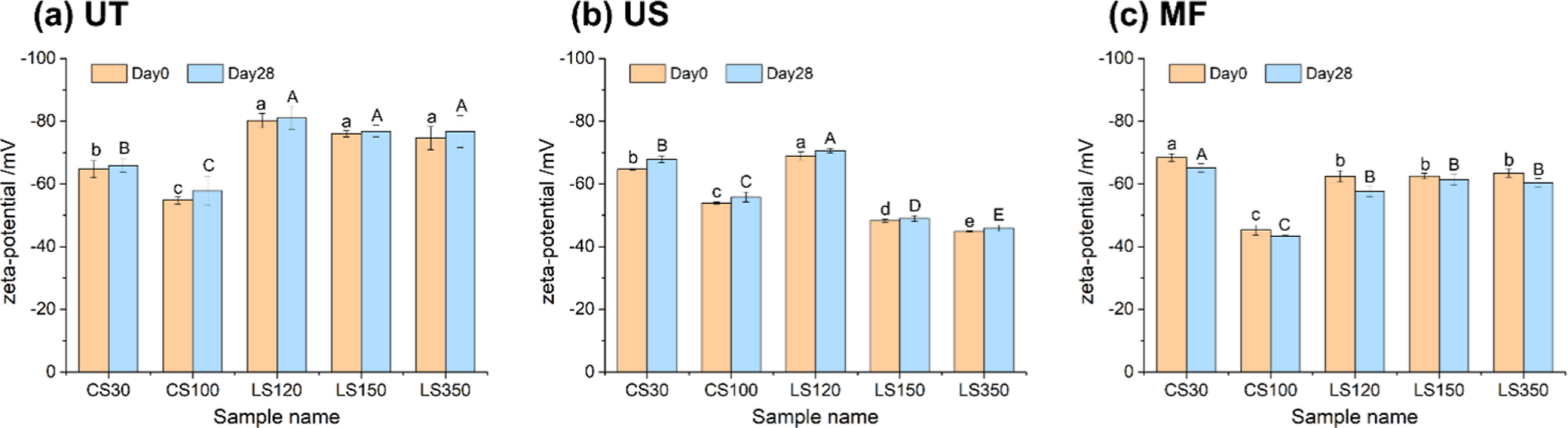
ζ-potential of Pickering emulsions with different processing methods and varied size of silica nanoparticles. Statistical comparisons were performed only among samples prepared using the same homogenization method. Lowercase letters indicate significant differences among emulsions at day 0, while uppercase letters indicate significant differences after 28 days of storage.

### Turbiscan Dispersion Stability

As discussed in Sections 3.2 and 3.3, Pickering emulsion stability arises from the combined effects of droplet size evolution and interfacial properties rather than a single parameter. To assess whether these structural and interfacial differences translate into long-term physical stability, turbiscan analysis was employed to monitor destabilization phenomena during storage.

The Turbiscan profiles (Figure 5) reveal that the destabilization mechanisms strongly depend on the processing method. For the UT prepared emulsions (Figure 5a,b), the decrease in backscattering at the very top region (>35 mm) is attributed to the presence of air bubbles immediately after preparation, which gradually dissipate during storage, leading to a reduction in the backscattering signal over time. The increase in backscattering at around 34–35 mm, together with a decrease in the middle region, indicates droplet creaming. This creaming process occurs rapidly, with a clear cream layer forming within the first 2 h. This behavior is consistent with the relatively large droplet size in UT prepared emulsions, which promotes faster gravitational separation. For the US- and MF-prepared emulsions (Figure 5c–f), the backscattering profiles show an increase at the top and a decrease throughout the bulk, indicating that droplet creaming is the dominant destabilization mechanism. Across all systems, the overall shape of the backscattering profiles remains relatively consistent over time, with no abrupt local changes. This indicates that destabilization is primarily driven by migration processes rather than droplet growth. At the same time, a slight increase in signal at the bottom suggests sedimentation of nonadsorbed particles, pointing to a combination of creaming and particle migration.

**Figure 5:**
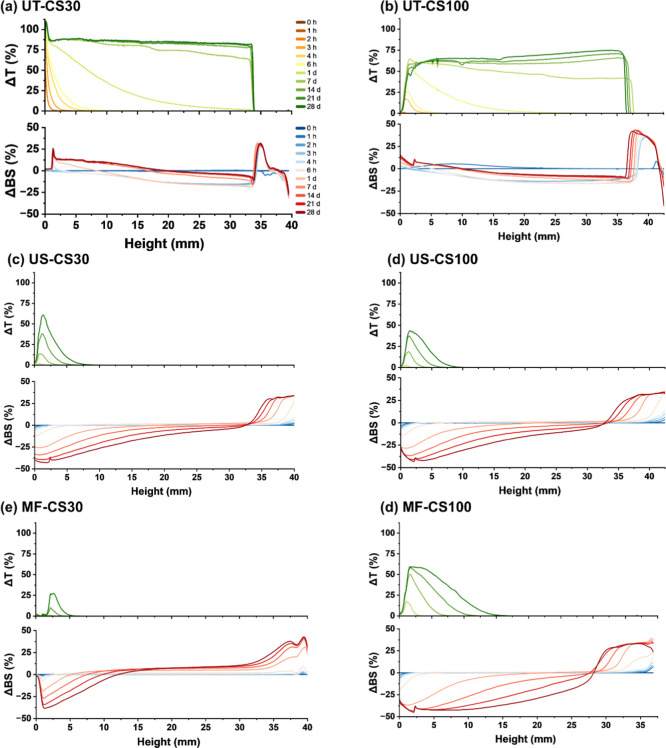
Transmission (ΔT) and backscattering (ΔBS) profiles for emulsions prepared by different homogenization methods: (a) UT-CS30, (b) UT-CS100, (c) US-CS30, (d) US-CS100, (e) MF-CS30, and (f) MF-CS100.

As shown in Figure 6a, UT-prepared emulsions exhibited rapid creaming within a few hours and clear phase separation after 1 week, whereas emulsions prepared by US and MF showed no visible oil layer throughout storage. The corresponding TSI values were substantially higher for UT emulsions than for US- and MF-prepared systems, indicating an inferior stability. This behavior is consistent with the larger droplet sizes and broader size distributions observed for UT emulsions, which promote gravitational separation. In contrast, the smaller and more uniformly distributed droplets produced by US and MF reduced the creaming and resulted in lower TSI values.

**Figure 6:**
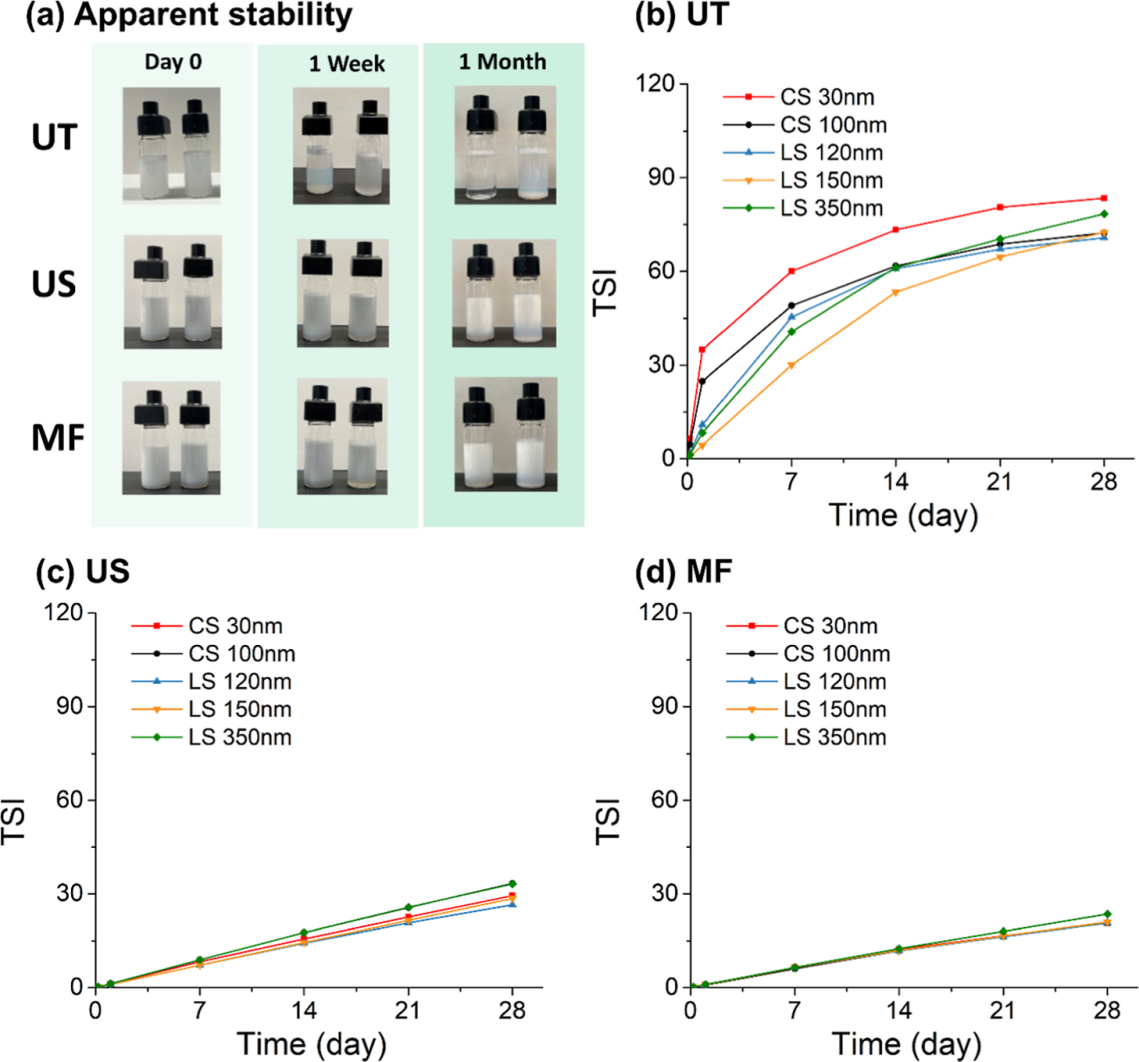
Physical stability of Pickering emulsions prepared with different methods and stabilizers. (a) Apparent stability of emulsions with CS30 and CS100. (b) Turbiscan stability index (TSI) of UT emulsion. (c) TSI of US emulsion. (d) TSI of MF emulsion. Please refer to Table 1 for the sample code.

The influence of the silica nanoparticle size on emulsion stability was strongly dependent on the homogenization method. For UT-prepared emulsions (Figure 6b), systems stabilized by intermediate-sized nanoparticles (CS100, LS120, and LS150) exhibited TSI values lower than those of systems containing smaller or larger particles. This trend reflects a balance between droplet size reduction, which favors stability, and particle sedimentation, which becomes more pronounced for larger nanoparticles. In contrast, for US-prepared emulsions (Figure 6c), particle size effects were relatively modest with slightly improved stability observed for smaller nanoparticles. For MF-prepared emulsions (Figure 6d), comparable TSI profiles were observed for particle sizes between 30 and 150 nm, whereas emulsions stabilized by 350 nm nanoparticles exhibited a higher instability. Overall, the Turbiscan results confirm that Pickering emulsion stability is governed by multiple interrelated factors, highlighting the need for multivariate analysis to identify the dominant factors controlling the emulsion stability.

### Multivariate Analysis of Factors Governing Emulsion Stability

PLS regression was applied to identify key factors affecting Pickering emulsion stability, using the Turbiscan Stability Index (TSI) at day 28 as the response variable. Predictor variables included particle size, droplet size descriptors (D­[4,3], D­[3,2], Dv(10), Dv(50), and Dv(90)), specific surface area of emulsion droplets, ζ-potential, and silica nanoparticle adsorption at the oil–water interface. The results of PLS analysis are shown in Figure 7.

**Figure 7:**
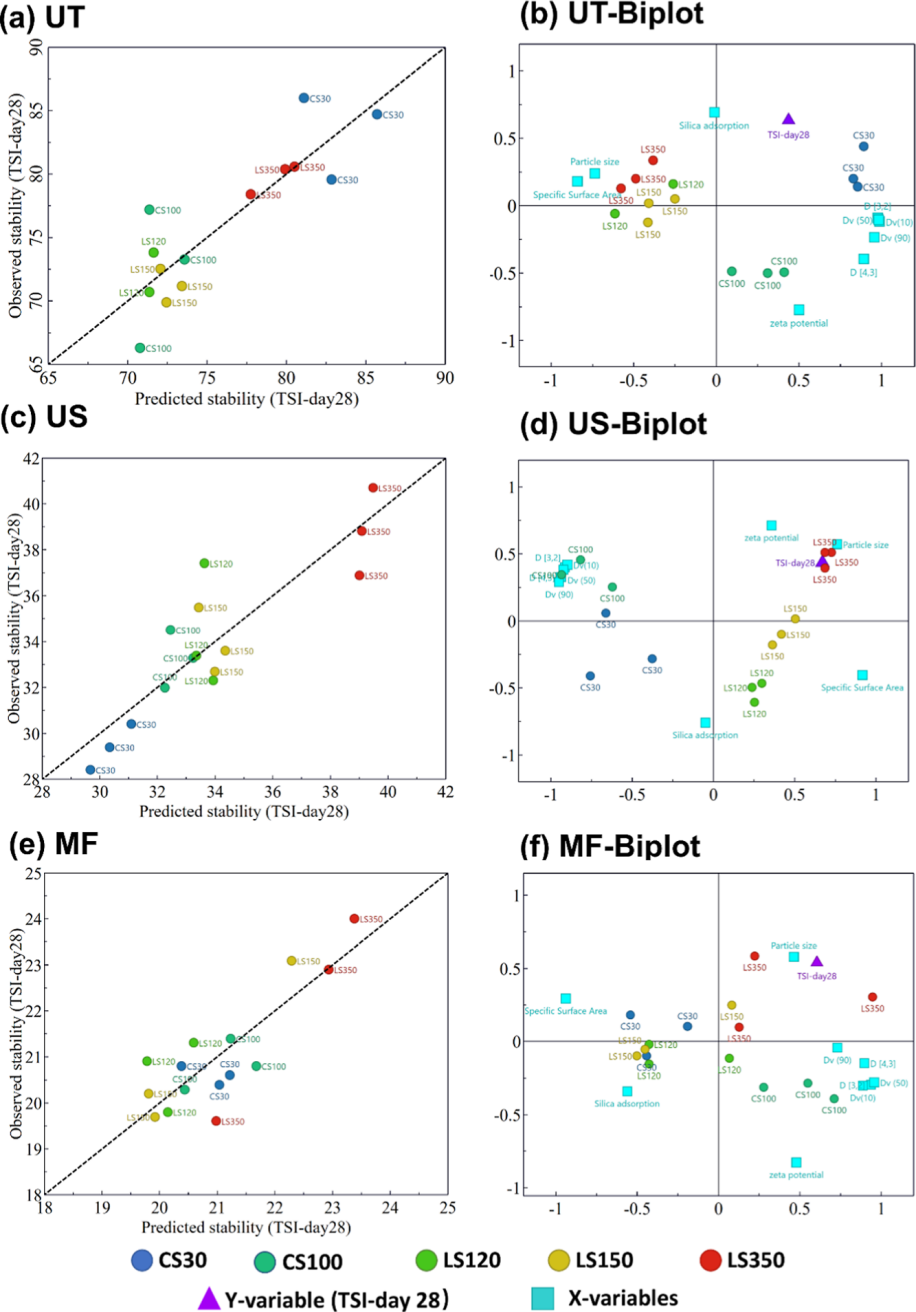
PLS regression analysis. (a,c,e) Predicted vs observed stability for UT, US, and MF emulsions, showing the model performance and correlation between particle size and emulsion stability. (b,d,f) Corresponding biplots illustrating the multivariate relationships among particle size, droplet size, silica adsorption, ζ-potential, and emulsion stability.

For UT-prepared emulsions, the score and loading plots (Figure 7a,b) show that emulsions stabilized by intermediate-sized nanoparticles (CS100, LS120, and LS150) clustered with lower TSI values and higher stability, whereas emulsions containing CS30 and LS350 were associated with lower stability. This behavior reflects a balance between competing mechanisms under UT conditions. Increasing particle size reduced droplet size, which slowed creaming and droplet coalescence, thereby improving stability. At the same time, larger nanoparticles were more prone to sedimentation due to gravitational effects, which contributed to an increase in TSI. As a result, emulsions stabilized by nanoparticles around 100–150 nm exhibited higher stability, whereas both smaller and larger particles led to reduced stability. These results indicate that, under low-energy homogenization, emulsion stability is governed by the interplay between droplet size evolution and particle sedimentation rather than by particle size alone.

For US-prepared emulsions (Figure 7c–d), TSI was positively correlated with particle size and negatively correlated with interfacial adsorption. These correlations indicate that, under ultrasonication, smaller silica nanoparticles promote emulsion stability primarily by enhancing adsorption at the oil–water interface. The high energy input provided by ultrasonication facilitates efficient particle adsorption, allowing smaller nanoparticles to achieve higher surface coverage and more effective interfacial packing. As a consequence, emulsions stabilized by smaller particles exhibited stronger resistance to droplet coalescence and creaming. This finding is consistent with the adsorption results discussed in Section 3.3 and confirms that, under US conditions, interfacial particle coverage is a key determinant of emulsion stability.

For MF-prepared emulsions, particle size and ζ-potential were identified as the most relevant contributors to emulsion stability (VIP > 1). The score plot (Figure 7e,f) shows that emulsions stabilized by nanoparticles in the size range of 30–150 nm exhibited similar stability, whereas emulsions containing 350 nm nanoparticles were associated with higher TSI values. Under MF conditions, the influence of particle size on stability was largely masked by limited coalescence during storage, as discussed in Section 3.2. Although high-pressure homogenization initially generated small and uniform droplets regardless of particle size, larger nanoparticles were more susceptible to sedimentation over time, which contributed to increased instability.

The PLS models show limited predictive ability (Q^2^ = 0.49, 0.56, and 0.33). This is likely due to the small data set and the inherent complexity of Pickering emulsions. Future improvements may be achieved by expanding the data set, incorporating additional variables, and applying more advanced modeling approaches, such as nonlinear methods. Despite this limitation, the PLS analysis indicates that Pickering emulsion stability arises from multiple interrelated factors, whose relative importance depends strongly on the homogenization method. Under UT conditions, stability is governed by the competing effects of droplet size reduction and particle sedimentation. Under US processing, enhanced interfacial adsorption associated with smaller nanoparticles dominates stability. In contrast, MF processing reduces particle size–dependent differences in droplet structure, and stability is primarily constrained by limited coalescence and particle sedimentation. These results provide a unified multivariate framework that rationalizes the complex stability behavior observed across different processing conditions.

## Conclusions

This study demonstrates that the influence of silica nanoparticle size on Pickering emulsion stability is strongly dependent on the homogenization method. Rather than exerting a universal effect, particle size governs emulsion stability through different mechanisms under different processing conditions. Under rotor–stator homogenization, emulsion stability was determined by a balance between droplet size reduction and particle sedimentation, resulting in optimal stability for intermediate-sized nanoparticles. Under ultrasonication and high-pressure homogenization, particle size effects on droplet structure were largely masked, and emulsion stability was mainly constrained with interfacial adsorption and sedimentation of larger particles. Overall, these findings indicate that particle size does not exert an intrinsic or monotonic effect on Pickering emulsion stability. Instead, its role is governed by the interplay among hydrodynamic stress, particle transport dynamics, interfacial adsorption, and gravitational settling within specific processing environments. We therefore propose a processing–particle coupling framework, in which stabilization behavior arises from the combined effects of droplet formation pathway and particle physicochemical properties. This mechanistic perspective provides guidance for the rational design of particle-stabilized emulsions and underscores the necessity of considering droplet generation conditions when evaluating particle size effects in colloidal systems. A limitation of the present study is that, although the same oleic acid modification protocol was applied to all silica particle sizes, the actual degree of surface modification varied because of differences in specific surface area and adsorption behavior. Consequently, the observed emulsification performance may reflect the combined effects of particle size and wettability. Future studies employing more precise surface functionalization and characterization techniques would help distinguish these effects more clearly and provide further insight into the role of particle size in emulsion stabilization.

## Supplementary Material



## Notes

Interfacial tension (IFT) of different dispersions against hexadecane; contact angle measurements of modified silica nanoparticles before and after ultrasonication and high-pressure homogenization; and AFM images of emulsion droplets (PDF)
