# Supplementary material for: Effect of Particle Size on Pickering Emulsion Stability Under Different Homogenization Methods
Source: Langmuir. 2026 Jun 15;42(25):17897–907. doi: 10.1021/acs.langmuir.6c01064 (PMC13325863; doi:10.1021/acs.langmuir.6c01064)
Supplement: Supplementary file 1 [file la6c01064_si_001.pdf]

Supporting Information

Effect of particle size on Pickering emulsion stability under different homogenization methods

Lin Chen [a,], Patrícia Figueiredo [a], Alaa Mahran [b,c], Ville A. Lovikka [a], Jessica M. Rosenholm [b], Maarit H. Lahtinen [a], Kirsi S. Mikkonen [a,d,*]*

[a]Department of Food and Nutrition, Faculty of Agriculture and Forestry, University of Helsinki, P.O. Box 66, FIN-00014 Helsinki, Finland

[b]Pharmaceutical Sciences Laboratory, Faculty of Science and Engineering, Åbo Akademi University, Turku 20520, Finland

[c]Department of Pharmaceutics, Faculty of Pharmacy, Assiut University, Assiut, 71526, Egypt

[d]Helsinki Institute of Sustainability Science (HELSUS), University of Helsinki, P.O. Box 65, FIN-00014 Helsinki, Finland

Corresponding authors Email: lin.x.chen@helsinki.fi, kirsi.s.mikkonen@helsinki.fi

Number of pages: 4

Number of figures: 2

Number of schemes: 0

Number of tables: 1

Table of contents

Table S1. Interfacial tension (IFT) of different dispersions against hexadecane S2

Figure S1. Contact angles of modified silica nanoparticles after ultrasonication and high-pressure homogenization treatment S2

Figure S2. AFM micrographs of SiNP-stabilized emulsion S3

Table S1 Interfacial tension (IFT) of different dispersions against hexadecane.

Interface	IFT (mN/m)
Water-hexadecane	43.8 ± 0.5
Oleic acid dispersion-hexadecane	32.1 ± 0.8
Bare CS30 dispersion-hexadecane	42.7± 0.3
Bare CS100 dispersion- hexadecane	42.5 ± 0.3
Modified CS30 dispersion- hexadecane	35.0 ± 0.4
Modified CS100 dispersion-hexadecane	33.6 ± 0.3

Oleic acid modification under high-energy treatment

High-energy treatment may affect the adsorption of oleic acid and induce rearrangement. We measured the contact angles of modified silica nanoparticles after ultrasonication (30% amplitude, 2 min) and high-pressure homogenization (80 MPa, 4 cycles), using CS30 and CS100 as representative samples. As shown in Figure S1, no significant differences between contact angles before and after high energy treatment were observed, indicating that the oleic acid layer was largely retained during processing.

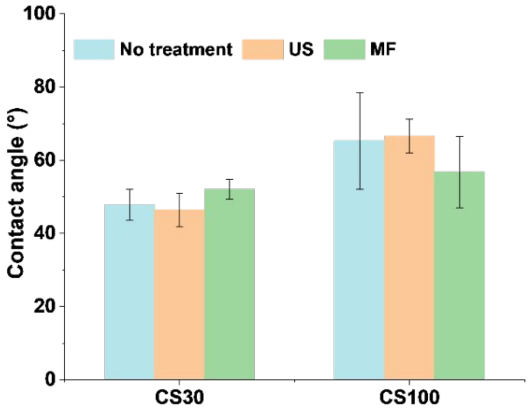

Figure S1. Contact angles of modified silica nanoparticles after ultrasonication and high-pressure homogenization treatment.

Emulsion observed by Atomic Force Microscopy (AFM)

AFM imaging was performed following the method reported by Lovikka et al.[1] Briefly, microscope glass covers were first plasma-treated (Zepto, Diener electronic, Germany) for 15 min and then immersed in an aqueous 1% polyethyleneimine (PEI)

solution for 1 h to reverse the surface charge from negative to positive. The covers were rinsed with Milli-Q water and dried with N_2 before use. The emulsion was then deposited onto the treated glass surface and allowed to stand for 15 min to enable droplet attachment. Excess emulsion was carefully removed until the liquid front approached the edges of the substrate. After sample preparation, the glass substrate was fixed at the bottom of a Petri dish using a small amount of grease. The dish was gently filled with Milli-Q water and transferred to an atomic force microscope (NanoWizard 4 XP BioScience, Bruker, USA) for imaging. AFM measurements were performed using silicon nitride cantilevers (MSNL, Bruker, USA) with nominal spring constants of 0.01–0.1 N/m. The cantilever stiffness was calibrated using the thermal excitation method. Imaging was conducted in QI Advanced mode. AFM data were processed using Gwyddion software.

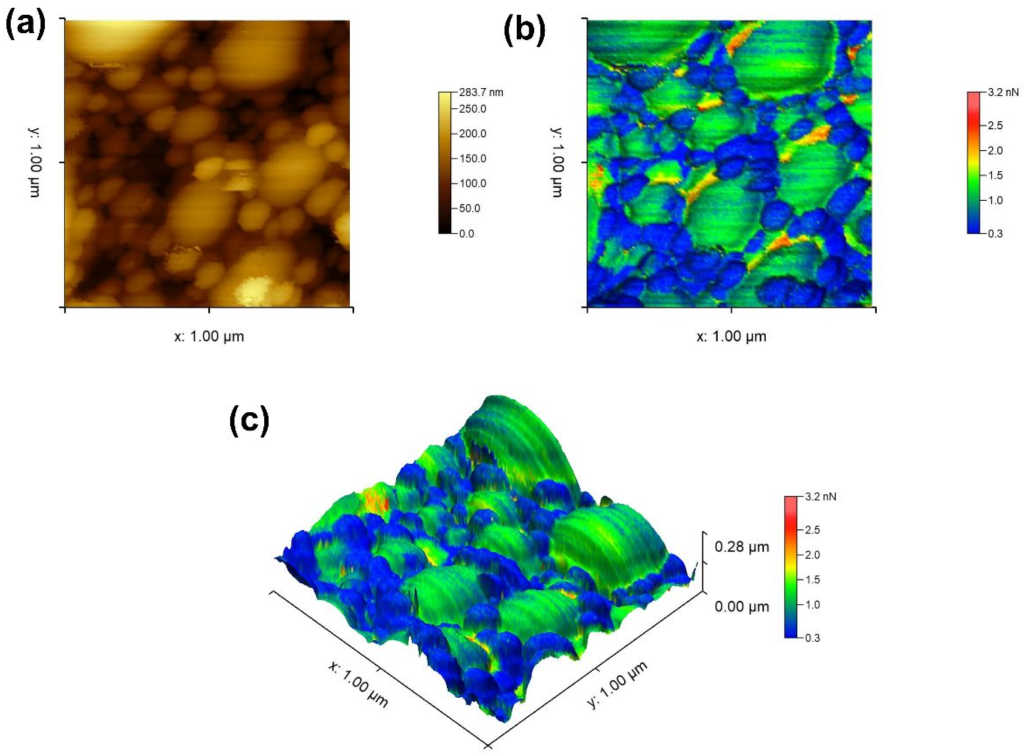

Figure S2. AFM micrographs of SiNP-stabilized emulsion. (a) Height image showing the morphology of emulsion droplets and interfacial nanoparticles. (b) Corresponding adhesion map highlighting contrast between droplets and particles. (c) Three-dimensional reconstruction of the surface topography.

The AFM results provide direct visualization of the droplet-particle relationship (Figure S2). During AFM measurements, silica nanoparticles and emulsion droplets exhibited markedly different adhesion signals. The topography image (Figure S2a) was colored according to the adhesion data, showing that silica nanoparticles display lower adhesion, while droplets exhibit higher adhesion (Figure S2b). This contrast can be attributed to differences in surface properties. Silica nanoparticles are relatively rigid and less adhesive under the measurement conditions, whereas the oil droplets are softer and more deformable, leading to higher tip-sample contact area and thus stronger adhesion signals. In addition, the interfacial structure was visualized more clearly by reconstructing the images in three dimensions (Figure S2c), which highlights the spatial relationship between silica nanoparticles and the droplet interface.

The AFM images reveal that the emulsion droplets (green) are larger than the adsorbed silica nanoparticles (blue). The nanoparticles are sparsely distributed at the interface, indicating incomplete surface coverage rather than a dense particle monolayer. These observations suggest that emulsion stabilization does not rely on full geometric coverage of droplets by rigid particles, but rather on partial adsorption and interfacial bridging.

Reference

(1) Lovikka, V. A.; Chen, L. ; Figueiredo, P. I.; & Mikkonen, K. S. Atomic Force Microscopy of Emulsions and Their Interfacial Nanoparticles. *Natural Sciences* **2025**, *5(3)*, e70016. https://doi.org/10.1002/ntls.70016
